# Surgery

**Published:** 1878-10

**Authors:** 


### Surgery.

Surgical Cases from a Phonographic Report of a Clinical Lecture. Lewis A. Sayre. (*Virgin. Med. Monthly.* May, 1878.)

Case I.—*Spondylitis of Cervical Vertabræ.*—This is the little girl whom you saw last Wednesday. She had been sick for two years, and had not walked for two months and a half. It is a case of spondylitis, or Pott's disease, effecting the cervical vertebræ. You remember we placed a plaster-jacket and jury-mast upon her last Wednesday; but after the lecture she complained of its uncomfortableness, which was sufficient proof that we did not do our work well. The jury-mast was placed a little too low down, so that its arch pressed upon the projecting portion of the spine. Dr. Vanderpool, the house surgeon, at once re-applied the jacket, raising the jury-mast a little, and now, as you see, she is able to walk without pain, though she is yet very weak.

This patient was brought in to illustrate the fact that mistakes in treatment are liable to be made even by those who have had considerable experience with similar cases, a fact which should serve as a warning to us when we are tempted for want of time, or other reasons, to dispatch patients too hastily. Whenever I make a mistake of this kind I learn something by it, and whenever it occurs in the treatment of my clinical patients, I bring them before you again, that we may learn a lesson together.

Case II.—*Chronic Inflammation of the Knee Joint—Amputation at the Joint—Mark's Artificial Leg—Hudson's Extensor.* —The patient who is now presented was first brought to this clinic a year ago. She has suffered since childhood from a chronic inflammation of the knee-joint. When only a few months of age she received an injury to the knee which gave rise to an inflammation that continued up to one year ago, at which time she was eleven years old. At that time her leg was luxated backwards behind the condiles of the femur and partially rotated outward. The deformity was due to reflex muscular contraction excited by the inflammation in the knee-joint. Numerous sinuses lead to dead bone, and from the exhausted condition of the patient, due to excessive discharges, no relief could be given except by exsection of the knee-joint or amputation. Exsection was out of the question, from the fact that the leg and foot had not been sufficiently developed to give the limb length enough to be of use, and therefore amputation was the only thing to be done. The question then came up, whether the amputation should be at the joint or in the shaft of the femur? Although several sinuses lead to necrosed bone in different portions of the femur, and one to the superficial portion of the patella, yet I decided to amputate at the knee-joint in preference to going through the shaft of the femur. From the exhausted condition of the child, I feared that section through the shaft would result in osteo-myelitis. The superficial necrosis of the femur, it was thought, could be successfully treated by the use of drainage tubes. I decided to perform Dr. Stephen Smith's operation, which, for the knee-joint, is preferable to all other operations. It leaves the cicatrix entirely behind, while the patella, *in situ*, acts as a firm basis of support for walking. The result in this case, as you see, justifies my decision.

Mr. Marks has been so kind as to present this little girl with one of his artificial legs, so that she is now able to walk about with freedom and comfort. There is one deficiency with the leg which she wears—she cannot extend it; there is nothing to take the place of the quadriceps extensor muscle. To obviate this defect we will have one of Dr. Hudson's extensors applied to the leg, which consists of an elastic band on either side of the leg plying over a portion of a wheel representing the quarter of a circle.

CASE III.—*Chronic Inflammation of the Knee-Joint, Complicated by Fracture of the Femur.*—This is a case which has been previously presented to you, of inflammation of the knee-joint, complicated by a fracture of the femur. There was deformity with fibrous anchylosis, the deformity being partially due to the fracture of the femur at the epiphysis. The fracture occurred when the patient was four years of age, but it was not recognized, owing to the fact that the inflammation which immediately followed, involved the knee-joint to such an extent as to direct all attention to the joint. She was treated constitutionally for white swelling of the knee-joint for several years, and finally recovered with her leg flexed at an acute angle and rotated outward, which was her condition, if you remember, at the time of the operation nine weeks ago. At that time, after breaking up the fibrous anchylosis at the knee, we experienced some difficulty in re-fracturing the femur, but fortunately succeeded in breaking it at exactly the point of original fracture, and placing the limb in perfect position. Extention was *immediately* applied, and Dr. Cuddeback, my assistant, dressed the limb with a plaster-of-Paris bandage. Sufficient compression was made with a wet sponge over the lower third of the femoral artery to cut off a portion of the supply of blood to the part, as is my usual custom after *brisement forcé* of the knee-joint, thus materially lessening the chances of inflammation. At that time, I told you we would be satisfied if, with the limb in position, we succeeded in getting anchylosis both at the point of fracture and at the joint. No constitutional or irritative fever followed the great violence which we did to the joint, and this I attribute largely to the immobile dressing which was used, and to the partial occlusion of the artery. We removed the dressing three days ago, and found that perfect consolidation had taken place at the point of fracture; fortunately, however, things had not proceeded so far at the joint. We therefore gave it slight passive motion, which has been repeated every day since that time, and now, as you observe, she is capable of walking without support, and has considerable motion at the knee-joint, which can be materially increased by the faithful administration of the tincture of time and essence of patience, together with elbow grease and palm oil, technically called *massage.* If the pain produced by manipulation in such cases as this, lasts more than twenty-four hours, the manipulation has been too severe or too protracted.

On account of the atrophy which exists in the affected limb, making it a little shorter than the other, it will be requisite to place about two thicknesses of sole-leather upon the bottom of the shoe worn on this foot in order to equalize the length of the two extremities. By so doing she will be able to walk without limping.

CASE IV.—*Double Talipes Equino-Varus with Luxation of Knee.*—Dr. Forest, of this city, has kindly brought a very interesting case of double talipes equino-varus with luxation of the knee. There is paralysis of the posterior muscles; and the quadriceps extensor contracting, bends the knee-joint exactly opposite to the normal direction. The deformity thus resulting resembles in fact, what it was first thought to be, a fracture of the epiphysis of the lower extremity of the femur.

We will seize the limb and bend the knee out in the proper direction. When we attempt to bring the *foot* into its normal position, before the deformity has been completely reduced, the foot becomes white, due to the obstruction to the circulation. If the foot were retained in this position, it would slough; if left alone, it would return to its abnormal position and become more and more crooked. The rule is, after manipulation of the foot, to retain it in a position as nearly normal as possible without interfering with free circulation. For the fixation of the foot, adhesive plaster or sole-leather dipped in cold water may be used. Great stress should be placed upon the thorough and careful manipulation of the deformed foot, to be repeated as often as possible by the attendants, for there is no mechanical appliance which can be substituted that has the adaptability of the human hand. I have now, during the short space of time which the patient has been before you, manipulated both feet to such an extent that I can place them in very nearly their normal position, where they will be retained to await further manipulation.

Case V.—*Result of a Case of Exsection of the Hip-Joint.*—George Brown, the young man now presented to you, is twenty-four years of age. When six years old, he received an injury to his hip from a fall. This gave rise to an inflammation in the hip-joint of a chronic character. Sinuses were formed on the outer and upper portion of the thigh, which discharged freely, and the patient became reduced to a skeleton. I removed the head, neck and trochanter major, and gouged the acetabulum on January 28, 1865, in this room. He was placed in the wire breeches, and removed to his home on the same day of the operation. He has recovered, as you see by his running up and down the steps and walking about the amphitheatre, with almost perfect motion. There is about one-half inch shortening. He is a large, strong and vigorous man, capable of doing a full day's work upon the farm, and has no inconvenience whatever from the limb.

Case VI.—*Necrosis and Abscess of the Tibia.*—This man had the lower end of his right tibia removed seven years ago. He has been walking about since that time, but has never completely recovered. In all probability, there is dead bone within. This is one of those cases which Dr. Markoe describes as *progressive sinuous abscess of the bone.* That the pus burrows its way along, is simply due to the fact that the abscess has not been opened at the most dependent point. If the opening be made at the lowest point at first, no further burrowing will take place.

In this case we have a pocket which retains the pus, the pus burrowing its way through the tissues. We will now proceed to drill through the bottom of this sac, and place therein a drainage tube, which will prevent the possibility of the retention of the products of inflammation, until nature has had time to exfoliate the dead bone; in the meantime, the patient can attend to his business without any difficulty whatever. The process of exfoliation may sometimes be hastened materially by the prompt removal of the greater portion of the dead bone, where the necrosis is extensive.

Case VII.—*Necrosis and Chronic Inflammation of the Tibia.*—This man had a portion of his tibia removed twenty-two years ago, but there was a little dead bone left which had given rise to an inflammation of a chronic character. One month ago, his leg measured fifteen inches in circumference; it now measures twelve and a half inches in circumference. I drilled holes through the leg and put in tubing, which was followed by a pretty sharp hæmorrhage. There is now some motion. The great point in the treatment of these cases is to establish free drainage in a downward direction, and thus prevent the retention and absorption of pus. If you have a free external outlet for the pus which is formed within, there is no trouble. Get rid of all the dead bone if you can by mechanically removing it; if not, get rid of a portion of it, and see that there is free drainage. By probing, I find that there is a good-sized piece of dead bone which could be removed with advantage to the patient, and thus save time and materially hasten the process of exfoliation. The external opening which leads to this dead bone should be dilated with a sponge-tent, after which, with the forceps, this large, necrosed portion may be quickly removed. The other portions of dead bone may be easily removed by picking them to pieces with a silver probe. We have now succeeded in drilling a hole through the bone, and after passing this large probe, will draw a drainage tube through the opening thus made, which will afford a free outlet to the pus.

CASE VIII.—*Sacro-Iliac Disease—Differential Diagnosis from Hip-Joint Disease.*—I now present you with a case which was sent to me through the kindness of Dr. Hunt, of New Jersey, and which was supposed to be a case for exsection of the hip-joint. Dr. Hunt first saw the patient (colored) two weeks ago, but no satisfactory history of the case has been obtained. All the information which I have been able to obtain has been from the patient himself since his arrival at the hospital. He states that he is eighteen years of age; that two years ago he suffered from what might be called a general rheumatic fever. He has been exposed to wet, but under what conditions and to what extent was not ascertained.

As yet no satisfactory diagnosis has been made, and it is for the purpose of arriving at some definite conclusion in reference to this interesting and obscure case, that I have brought it before you.

When I first saw him three days ago, he was sitting up in bed, and occupied the exact position which a patient in the second stage of hip-joint disease usually occupies; that is, with the leg flexed upon the thigh, the thigh upon the trunk, the foot everted, and the limb *abducted.*

We will now apply Nélaton's test for displacement of the head of the femur, which consists in passing a line from the anterior-superior spinous process of the ilium to the tuberosity of the ischium. A line thus drawn will pass, when there is no displacement, exactly over the apex of the trochanter major, which we find to be the course of the line in this case. Now, if we had a fracture of the neck of the femur, the trochanter would be above this line; if absorption of the neck, head or acetabulum, the trochanter would also be above this line; the leg would be *adducted,* inverted and shortened. The position which the patient occupied when I first saw him, was sitting with his left leg flexed, abducted and rotated outward, which is the position of a limb when the hip-joint is over-distended, as with pus or serum. Further, in hip-joint disease, if the capsule of the joint be not ruptured, you cannot invert the toe, adduct the limb, or extend the thigh, without producing a great deal of pain. But in this case, taking care not to affect parts external to the joint by the motion of the limb, and to hold the pelvis absolutely still, we can invert the toe, and slowly adduct and extend the limb without causing pain. This would go to show that it was not the hip-joint which was involved, or else that the capsule of the joint has been ruptured, and the effusion into the joint squeezed out. Yet this can hardly be the case, for as we look at the limb, we see that it occupies the position common to the *second* stage of hip-joint disease. If the capsule of the joint had been ruptured or perforated so as to liberate the pus, the limb would no longer retain its present position, but would assume the position common to the *third* stage of hip-joint disease. Now, the limb can get in this position from muscular contraction, but if the deformity be due to muscular contraction, we can invert the leg and rotate it in, which movements we find can be effected in this case. This fact alone has satisfied me that there is no distention of the joint.

You probably will have observed that in making our examination we have avoided, ar far as possible, all sources of irritation and excitement to the patient, for when these patients become irritated, it is almost impossible to do anything with them. As he lies quietly on his couch, we may make some further observations. Our object is to elicit the exact location of the disease. By the application of Nelaton's test line, we have found that the trochanter major is in its normal position. I can crowd the head of the femur firmly into the acetabulum without causing pain. And, again, the leg is too long, and its position too good for a carious head of the femur, or carious acetabulum. You will observe that so long as we confine our examination to the hip-joint proper, our manipulations give rise to no pain; but when we crowd the ilii together with only a slight force, great pain is produced. By passing my thumb around the sacro-iliac junction, I elicit severe pain, and upon the inside I find a fullness which is indicative of a sacro-iliac abscess which has burrowed its way along down the thigh. In my examination of the patient three days ago, the pus broke out on the left side at the upper portion of the thigh. Nearly a half pint escaped, and so strongly was it impregnated with the odor of fæcal matter, that at first I took it to be a portion of the contents of the large intestine, but further examination proved this not to be the case. The matter discharged must have lain for a long time in contact with the rectum, and have derived its odor from the fæces through the principle of osmosis. I am happy to state that Dr. Stephen Smith agrees with me as to the probability of this explanatory conjecture.

It is painful to witness the extreme suffering of this man ; but to-day he is far more comfortable than when I first examined him.

Many of the symptoms which we have elicited from this patient this afternoon are present in cases of hip-joint disease, but it has been our aim to show you how they are to be differentiated from those of true hip-joint disease. If I make firm pressure over the iliac fossa on the right side I get no pain, but pressure in the same situation on the left side gives rise to extreme pain. Now observe what I am doing. Extension of the femur gives the patient ease; so it does in hip disease, but you will observe that I am extending the ilium through its attachments to the femur. I am making extension upon the sacro-iliac junction, and that is where the disease exists. As soon as I cease to make extension the patient is in agony, but so long as the extension is applied he is at ease. By placing my left hand over the superior crest of the ilium and pulling with great force, thus drawing the ilium from the sacrum, great relief is afforded the patient. While holding the ilium away from the sacrum, I am crowding the head of the femur firmly into the acetabulum, which gives rise to no pain, and settles the question that there is no disease of the hip-joint.

We have spelt out this case, as it were, and can now easily arrive at a correct diagnosis. We have found in our examination that so long as we do not affect parts external to the hip-joint, our manipulations give rise to no pain. With the sacro-iliac articulation extended, we can make firm compression over the trochanter major without producing pain. When the ilia are crowded together, intense pain is produced. Here, then, we have a clear case of *sacro-iliac disease*, which has gone on to suppuration, and the pus has found its way along down under Poupart's ligament, and come out upon the anterior portion of the thigh.

With reference to the treatment of this case, the first step of essential importance is that a free outlet for the imprisoned pus should be made, and the parts cleansed thoroughly. For this purpose, we will make a free incision along the anterior portion of the thigh were the pus has accumulated, rinse the cavity out with carbolic wash, fill it with Peruvian balsam, and stuff in some oakum. Had the abscess opened posteriorly over the sacro-iliac articulation, I would have made a free incision down to the joint, and laid open fully any sinuses leading to dead bone, removing at the same time any accessible portions of necrosed bony tissue. By placing this patient in the upright posture, the chances for drainage will be greatly improved, and the antiseptic applications which we have made will tend to bring about a more healthy state of affairs.

In the treatment of these cases of sacro-iliac disease, I have recommended extension. During the time when the patient is in the erect posture, extension is to be made by increasing the thickness of the sole of the shoe which is worn on the foot of the unaffected side to such an extent as will permit the foot of the affected side to swing clear of the ground, and thus extension upon the sacro-iliac articulation will be made by the weight of the limb on the affected side. The extending force may be further increased by running lead into the sole of the shoe on the affected side. This method of extension is intended only to be used while the patient is exercising on his crutches. At night, and whenever resting in the horizontal posture, extension is to be kept up by a weight and pulley over the foot of the bed. The foot of the bed is to be raised a few inches higher than the head of the bed, by means of which the body acts as a counter-extending force. In my work on *Orthopœdic Surgery*, I have probably not been as explicit with reference to this part of the treatment as I should have been, and therefore some persons have imagined that I intended to keep the patient in the erect posture day and night until recovery took place. But I there stated that the same principle of treatment has to be carried out in dealing with sacro-iliac disease as was used in the treatment of hip-joint disease, which implied extension and counter-extension by means of a weight and pulley over the foot of the bed whenever the patient was lying down. However, I have not been understood, for Dr. McGuire, in the *Virginia Medical Monthly* for January, 1878, has thus misunderstood me. The abscess of the anterior portion of the thigh is now opened freely, and a large amount of very offensive pus with a distinct fæcal odor is escaping.

I have just made out another important point in this case. By introducing my finger through the incision which has been made to liberate the pus, I can pass it up and about the capsule of the joint which I find to be unruptured. This confirms our exclusion of the hip joint from disease.

We now inject the cavity with carbolized water, and having thoroughly washed it out, fill it with Peruvian balsam. We elevate the limb, so that the disinfecting balsam may come freely in contact with all parts internally. We will stuff in some oakum, cover the part with oil silk, and over this apply a roller bandage. Extension must be made as we have already suggested. The patient should have a pair of crutches, so that he can get out into the open air and sun-light. He should have a sustaining diet.

Removal of Gravel from the Bladder.—By M. Dubuc. (*L'Union Médicale*, July 23, 1878.) M. D. presented to the Société de Médecine de Paris specimens of gravel—uric acid crystals—which the patient had been able to extract himself. He was a man 80 years of age, who had been operated upon—lithctrity—in 1867 and again in 1876. Convinced that he had to expect a renewal of the difficulty unless some means were adopted to prevent the accumulation, he, the patient, resolved to use aspiration in thoroughly emptying the bladder.

In 1877, M. D. exhibited to the society some uric acid crystals thus removed that were four millimeters in diameter. He exhibited, on the occasion in question, about fifty in number, and some of them six millimeters in diameter. The patient had not suffered from an attack of renal colic for more than twenty-five years, although he was constantly passing these uric acid crystals. He was in the enjoyment of excellent health.

The aspiration was at first practiced once a day, and then gradually reduced to once in six days. The instrument used was simply a glass syringe.

In the course of discussion on this subject, M. Mercier described the following method of accomplishing the same work. Previous to micturition the patient is requested to lie flat on his belly. After a few minutes of repose in this position, he rises slowly on all fours, and whilst in this position, is requested to pass his water. The object is to remove by their specific gravity the gravel from the cul-de-sac and force them along with the current.

Plaster of Paris Dressing for Transverse Fracture of Patella.—Reported by M. Droullon, service of Dr. Denis-Dumont. (*L'Année Médicale, July*, 1878.)

The history of the case which gave use to the following plaster of Paris dressing runs thus:

Mr. V., a railroad worker, received a kick on the 28th of May,

1876, in the front part of right knee; began to run to avenge himself but found that he was helpless, not being able to extend his right leg. He went to the Hospital Lisieux; a dressing of dextrine was applied and on the 4th of September, 1876, he left the hospital cured.

He then walked, in order to get home, twenty-eight kilometers. In the evening, without any perceptible sudden pain, he perceived that the formerly united fragments were again separated, and he was utterly unable to carry the leg forward.

A fortnight later, 20th of September, he entered the Hôtel-Dieu de Caen. In consideration of the history it was thought the union would be accomplished with difficulty, and the following plaster of Paris dressing was devised.

Materials used: Two bands of tarlatan—twenty-four thicknesses—six centimeters wide—eighty long. Two pieces of strong tape two centimeters wide and fifty long. A steel buckle attached to a rubber strap from fifteen to twenty centimeters long. Half a litre of plaster of Paris.

Mode of application: The plaster being properly mixed, one of the bands of tarlatan is well saturated with it and placed round the lower part of the thigh—the leg being slightly flexed—so as to form a ring, placed obliquely from before backwards, and from above downwards; the lower border of the ring must adapt itself well to the upper border of the upper half of the patella, whilst behind, it passes down obliquely into the popliteal space. Whilst adapting this band the two pieces of strong tape must be well inserted between the folds of the tarlatan one in the middle behind, so that the free end may hang down towards the leg; the other similarly situated in front.

The second band is prepared in much the same way. The direction, however, must be different, namely the obliquity will be from below upwards and from before backwards. The upper part of the band must be snugly adapted to the lower border of the lower half of the patella. During its application the loose piece of tape, in the upper ring behind, must be incorporated in the band, and the buckles attached to the rubber bands must be inserted in front just opposite the center of the lower border of the patella and opposite the tape inserted in the upper ring.

The plaster being dry, the leg is extended and the two fragments are brought slowly together, the distances being slowly decreased according to the judgment of the surgeon.

In the case in question, the parts were not brought together until the fourth day. On the 15th of October the parts had somewhat diminished in size and a fresh application was made. This remained in place till the 10th of February, 1877.

The patient was discharged on the 14th of March with a slight stiffness in the joint. When seen in December last, all traces of the accident had disappeared.

Treatment of Erysipelas by Hypodermic Injections of Carbolic Acid. By Herman Hueter. (*Berl. Klin. Wochenschrift*, No 24, 1878.)

At the surgical clinic in Greifswald, subcutaneous injections of carbolic acid in traumatic erysipelas have been in use during the past year. In order to get along with the least quantity of the drug, great care is taken to attack the disease in the beginning. After every febrile attack in cases of wounds the parts are kept under observation and within some hours the diagnosis of erysipelas is usually established by a reddening of the edges of the wound. The wound is now thoroughly cleansed with carbolic acid, or if the appearance is unfavorable, with a five to eight per cent. solution of chloride of zinc. The solution used for hypodermic injections consists of carbolic acid and alcohol, ana 1.5, water 50 grams. One syringeful (1 C. C.) of this fluid diffuses itself under the skin to the extent of about one half the size of an ordinary card. For repeated injections the needle may be left in place, since the solution spreads very easily. While ordinarily three to eight C. C. have sufficed, Hueter has injected as much as twelve syringes without accident. By surrounding the entire border of the erysipelatous patch with a ring of fluid in the subcutaneous tissue, a further progress of the disease is at once checked. If the patch is large, a second series of injections within its borders, may be necessary, whereupon a speedy return to the normal pallor of the skin shows the disappearance of the disease. No better test of the efficacy of this method could be desired, than the repeated observation, that where the points of injection were too far apart, the erysipelatous inflammation continued to extend in a peninsular shape between the protected portions.

Where red, hard cords indicate the existence of a lymphangitis, a thorough application of mercurial ointment along their tracks was found most servicable. For internal treatment there is rarely any call for this method.—*Allg. Med. Central Zreit. No.* 53-54-55, 1878.

